# Short-term organic fertilizer substitution increases sorghum yield by improving soil physicochemical characteristics and regulating microbial community structure

**DOI:** 10.3389/fpls.2024.1492797

**Published:** 2024-11-08

**Authors:** Mengen Nie, Guangqian Yue, Lei Wang, Yizhong Zhang

**Affiliations:** ^1^ Center for Agricultural Gene Resources Research, Shanxi Agricultural University, Taiyuan, China; ^2^ College of Agronomy, Shanxi Agricultural University, Taiyuan, China; ^3^ Shanxi Key Laboratory of Sorghum Genetic and Germplasm Innovation, Sorghum Research Institute, Shanxi Agricultural University, Jinzhong, China

**Keywords:** organic fertilizer, microbial community, physicochemical factors, biomarkers, sorghum

## Abstract

**Introduction:**

Chemical fertilizer reduction combined with organic fertilizer (organic fertilizer substitution) has a positive impact on crop productivity and sustainable development. However, the effects of short-term organic fertilizer substitution on microbial community structure and functions of sorghum rhizosphere soil and on sorghum yield remain unclear. Herein, this study investigated the short-term effects of organic fertilizer substitution on sorghum soil physicochemical properties, microbial community structure and enzyme activities using Metagenomic sequencing technology.

**Methods:**

The fertilization treatment included no fertilization (CK), pure chemical fertilizer N (CF), substitution of 25% chemical fertilizer N with organic N (NF25), substitution of 50% chemical fertilizer N with organic N (NF50), substitution of 75% chemical fertilizer N with organic N (NF75), and pure organic fertilizer N (NF100); soil samples were collected and analyzed in the flowering period of sorghum.

**Results and Discussion:**

The results showed that the suitable organic fertilizer substitution rate of sorghum field was 50%, and its yield was the highest among all treatments (9789.9 kg/hm^2^). Compared with the CF treatment, a medium ratio (50%) of organic fertilizer substitution significantly reduced soil alkalization (by 3.05%), improved soil nutrients, enhanced soil enzyme activities, and increased sorghum yield (*P* < 0.05). After organic fertilizer substitution treatment, higher protein, fat, and total starch levels accumulated in sorghum grains, and the tannin content of grains decreased. The effect of organic fertilizer substitution on bacterial diversity was greater than that on fungal diversity. Among the dominant bacterial phyla, the medium ratio of organic substitution treatment significantly increased the relative abundances of Proteobacteria (by 3.57%) and Actinomycetes (by 14.94%), and decreased the relative abundances of Acidobacteria (by 5.18%) and Planctomycetes (by 7.76%) compared with no fertilization, while the dominant fungal phyla did not respond significantly to the addition of organic fertilizer. Organic fertilizer substitution also improved soil microbial metabolic pathways, biosynthesis of secondary metabolites, and carbon metabolism. The biomarkers enriched in inorganic fertilizer treatment and organic fertilizer substitution treatments had similar relevant environmental elements but reversed correlation trends. Moreover, soil Alkali-hydrolyzable nitrogen and L-leucine aminopeptidase were important environmental factors influencing the structure of bacterial and fungal communities in sorghum soils, respectively. Soil nutrient levels and microbial communities together explained the variation in annual sorghum yield. The results of this study provide evidence that short-term organic fertilizer substitution increases sorghum yield by improving soil properties and regulating microbial community structure.

## 1 Introduction

Sorghum (*Sorghum bicolor* (L.) Moench), as one of the oldest cereal crops, plays an important role in Chinese brewing industry. Its grains are not only edible, but also widely used for brewing wine and vinegar (Dabija et al., 2021). In addition, soil salinization is a serious environmental issue in North China. Sorghum is a potential crop for production in saline soils because of its wide adaptability and high tolerance to drought, poor, and salinity stresses (Nie et al., 2023). In order to increase sorghum yield, a large amount of chemical fertilizers were applied to farmland. Excessive input of chemical fertilizers, especially nitrogen fertilizers, lead to a variety of environmental problems, such as the decrease of nutrient utilization efficiency, the acceleration of soil organic carbon loss, and the destruction of soil structure (Corato, 2020; Zhao et al., 2021). Organic fertilizers are rich in organic matter, beneficial microorganisms and a variety of nutrients needed for crop growth, which can improve the environmental problems caused by excessive application of chemical fertilizers (Chaudhary et al., 2022; Liu et al., 2021b). However, due to the slow release of nutrients from organic fertilizers, the single application of organic fertilizers cannot meet farmers’ demand for increasing crop yield in the current season (Cheng et al., 2021). Many studies have shown that chemical fertilizer reduction combined with organic fertilizer (organic fertilizer substitution) is an effective measure to maintain soil health and improve soil fertility and crop productivity (Cheng et al., 2021; Corato, 2020; Liu et al., 2021b; Lu et al., 2021). Lu et al. (2021) found that the organic nitrogen substitution rate of 18% to 24%, which could increase soil available nutrient contents and fertilizer utilization efficiency while achieving high and stable crop yields.

Organic fertilizer can comprehensively improve the negative impact of long-term excessive application of inorganic fertilizers on the soil quality and effectively improve crop productivity (Ji et al., 2020; Huang et al., 2023). Soil salinization in northern China is a serious problem. Humic acid and fulvic acid contained in organic fertilizers can neutralize the alkaline substances in the soil, and long-term application of high amounts of organic fertilizers in alkaline soils can effectively reduce soil pH (Guo et al., 2024a). Compared with the application of chemical fertilizers alone, the addition of organic fertilizers can reduce soil bulk density, providing good soil conditions for the storage of soil organic matter, nitrogen, and phosphorus (Corato, 2020). At the same time, the application of organic fertilizers is also conducive to the effective capture of N and P in the topsoil and prevent their leaching (Wang et al., 2020). Ren et al. (2021) observed that partial replacement of chemical fertilizer with organic fertilizer can not only increase the total amount of soil nutrients, but can also improve soil nutrients availability, and ultimately increasing maize yield. Soil enzymes are mainly derived from microbial metabolism and plant root secretions (Zhang et al., 2020). Liu et al. (2021a) showed that long-term organic fertilizer substitution for chemical fertilizer can increase the relative abundance of beneficial soil microorganisms and indirectly improve rice yield through soil enzyme activities and nutrients. In addition, the application of organic fertilizer also has a positive effect on crop quality. Guo et al. (2024b) found that long-term combined application of manure and chemical fertilizer increased protein content of winter wheat grains, and optimized the protein component characteristics of winter wheat grains.

Soil microorganisms play an important role in ecosystems, they participate in a variety of ecosystem processes, including nitrogen fixation, soil structure improvement, nutrient mineralization, nutrient cycling, and plant nutrient uptake and growth (Bardgett and van der Putten, 2014; Ji et al., 2020). Reasonable application of organic fertilizers can regulate the structure of soil microbial communities and improve the soil microbiological environment (Liu et al., 2021a; Zhang et al., 2020). Zhang et al. (2020) found that applying cow manure not only increased the diversity of soil bacteria and the relative abundance of beneficial microorganisms, but also effectively regulated the soil bacterial community structure in tea plantations. Ji et al. (2020) showed that the increasing proportion of organic substitution led to a more complex fungal co-occurrence network, and the complexity of fungal co-occurrence network was positively correlated with soil quality index. Long-term application of organic fertilizers also plays a certain role in improving the functions of soil carbon and nitrogen cycles (Lian et al., 2021; Liang et al., 2023). Previous studies mainly focused on long-term field trials (Ji et al., 2020; Liu et al., 2021a; Ma et al., 2022), although several studies have confirmed that short-term organic substitution has a significant effect on improving crop yields (Lazcano et al., 2013; Lian et al., 2021). However, the effects of short-term organic fertilizer substitution on the functional diversity of sorghum soil microbial communities and the correlation between soil microbial community diversity and environmental factors remain unclear. Therefore, it is of great significance to study the short-term effects of organic substitution on the physicochemical properties, microbial characteristics and enzyme activities of sorghum rhizosphere soil.

Previous studies have shown the effects of fertilization patterns on sorghum yield, soil physicochemical properties, and soil enzyme activities (Dong et al., 2017; Wang et al., 2023; Wang et al., 2019). However, the impact of changes in soil microbial communities and soil physicochemical properties on sorghum yield after short-term chemical fertilizer reduction combined with organic fertilizer is still unclear. Therefore, in this study, a short-term organic substitution experiment was carried out on soil applied with chemical fertilizer alone for 5 consecutive years in the Xiuwen Experimental Base of Shanxi Agricultural University. The flowering period is the most active stage of nutrient exchange between root and soil during the plant growth cycle (Tao, 2022). Therefore, we collected soil samples during the sorghum flowering period for metagenomic sequencing. The objectives of our research were (1) to investigate the effects of organic fertilizer substitution on sorghum yield, grain quality, soil physicochemical properties, soil microbial community structure and functions; (2) to explore the correlations between soil physicalchemical properties and microbial communities; (3) to explore the contributions of soil nutrients and microorganisms to the improvement of sorghum yield. This study aims to provide theoretical basis for rational fertilization and soil ecological environment improvement in sorghum fields.

## 2 Materials and methods

### 2.1 Experimental sites and experimental materials

The field experiment was conducted on May 5, 2022 at the Xiuwen Experimental Base of Shanxi Agricultural University (37°62′N, 112°71′E), which belongs to the typical semi-moist warm temperate continental monsoon climate. The average annual temperature is 9.8°C, the annual rainfall is 418-483 mm, the annual sunshine hours are 2,662 hours, and the frost-free period is 158 days. The tested soil type is brown soil. The basic properties of the topsoil (0-20 cm) are: pH 9.01, organic matter 11.2 g.kg^-1^, total nitrogen 0.82 g.kg^-1^, alkali hydrolyzed nitrogen 49.6 mg.kg^-1^, available phosphorus 35.3 mg.kg^-1^, and available potassium 230.6 mg.kg^-1^. The tested grain sorghum variety is ‘Hongnuo 16’.

### 2.2 Experimental design

The experiment design included six treatments: CK (no fertilization), CF (pure chemical fertilizer, 100% chemical fertilizer N), NF25 (low ratio of organic fertilizer substitution, 75% chemical fertilizer N + 25% organic fertilizer N), NF50 (medium ratio of organic fertilizer substitution, 50% chemical fertilizer N + 50% organic fertilizer N, NF75 (high ratio of organic fertilizer substitution, 25% chemical fertilizer N + 75% organic fertilizer N, and NF100 (pure organic fertilizer, 100% organic fertilizer N). The experiment was conducted in a randomized block design with three replications. Each experimental plot area was 48.0 m^2^ (16m × 3 m), with a row spacing of 50 cm and a plant spacing of 20 cm. All organic and chemical fertilizers were applied to the soil entirely before planting. The nitrogen, phosphorus, and potassium contents were the same in all treatments except CK, and the substitution rates for all treatments was based on the same nitrogen content, and inorganic fertilizer was added when phosphorus and potassium were insufficient. The total amounts of N, P_2_O_5_, and K_2_O applied were 225,140, and 140 kg/hm^2^, respectively. The chemical fertilizers applied in the experiment were urea (N 46%), superphosphate (P_2_O_5_ 16%) and potassium sulfate (K_2_O 50%). The organic fertilizers, namely, cow manure with 41.5% organic matter, 1.67% N, 0.43% P_2_O_5_, and 0.95% K_2_O (by air dried weight), which was purchased from Hebei Rundong fertilizer Co., Ltd., China. Sorghum was sown on May 16, 2022, with a row spacing of 50 cm, a plant spacing of 20 cm, and a planting density of 100,000 plants/hm^2^. Furthermore, Irrigation, pest prevention, and field weeding management employed local agricultural management practices.

### 2.3 Soil sample collection, sorghum grain yield and quality determination

Samples of rhizosphere soil (0-20 cm) were collected during the sorghum flowering period (growth stage 65 in Biologische Bundesanstalt, Bundessortenamt and Chemical Industry scale) on August 06, 2022). In each plot, five sorghum plants were uprooted using the five point sampling method, the roots were gently shaken to remove loose soil, and the residual soil was collected from the roots using a sterile brush and mixed into a composite sample (Ren et al., 2021). The collected soil samples were properly stored in the freezer and transported back to the laboratory as soon as possible. After removing impurities such as plant residues and stones through a 2 mm mesh, the rhizosphere soil was collected. Each sample was subsequently divided into two parts, one part was stored at -80°C to be used later for metagenomic sequencing, and the other was aie-dried in a cool and shaded area for the determination of the physicochemical factors.

In the harvest period of sorghum (growth stage 65 in Biologische Bundesanstalt, Bundessortenamt and Chemical Industry scale) on October 02, 2022, three square meters of sorghum ears were randomly selected for threshing in each plot to determine its yield. After the harvested grains were naturally dried for 2 weeks, the content of protein, total starch, fat and tannin were measured using a near-infrared grain quality analyzer (Infratec TM 1241, Foss, Denmark).

### 2.4 Soil physicochemical properties and enzyme activities

Soil pH and EC were measured using a pH meter (PHS-3C, Leici, Shanghai, China) and a conductivity meter (DDS-11A, Leici, Shanghai, China) under the condition of a soil to water ratio of 1:2.5, respectively (Bao, 2000). Soil bulk density (BD) and soil water content (SWC) were measured according to Bao (2000). Soil organic carbon (SOC) was determined by K_2_Cr_2_O_7_-FeSO_4_ oxidation method (Bao, 2000). Total nitrogen (TN) was determined by Kjeldahl method (KDN-102C, Shanghai, China), total phosphorus (TP) was determined through a UV-VIS spectrophotometer (U-2900, Hitachi, Japan) using molybdenum blue colorimetric analysis, and total potassium (TK) was measured via the flame photometry (FP6400A, Shanghai, China) (Bao, 2000). Alkali-hydrolyzable nitrogen (AN), available phosphorus (AP), and available potassium (AK) were measured by diffusion method, Olsen method, and ammonium acetate extraction method, respectively (Bao, 2000). Alkaline phosphatase (ALP), β-D-Glucosidase (BG), L-leucine aminopeptidase (LAP) and β-N-acetylglucosaminosidase (NAG) were determined using the kit method (BC0280, BC0165, BC4025, BC4005; Beijing Solaibao Technology Co., Ltd., Beijing, China).

### 2.5 Soil microbial metagenomic sequencing

Total microbial DNA was extracted from soil using an TGuide S96 Magnetic Soil/Stool DNA Kit (BeiJing, China). The integrity of the genomic DNA was determined by agarose gel electrophoresis, and the concentration and the purity of the genomic DNA were determined using TBS-380 and NanoDrop200, respectively. The sequencing Library was constructed with VAHTS Universal Pro DNA Library Prep Kit for Illumina kit (NanJing, China), and fragment detection and quantitative detection were performed with Qsep-400 and Qubit3.0 instruments. Illumina NovasqTM6000 platform by LC Bio Technology CO.,Ltd (Hangzhou, China) was used for Metagenomic sequencing of the qualified libraries. The raw data have been stored in the NCBI database under accession number PRJNA1149528.

Quality control and filter out of host contaminations were performed on the original sequences to obtain the final effective reads, which were assembled using the MEGAHIT software (https://GitHub/outscn/megahit), and contigs with a length greater than 500bp were retained for subsequent clustering analysis. Use MetaGeneMark (v3.26) software to perform the coding regions CDS prediction on the assembled contigs, and finally constructed non-redundant Unigenes sets using CD-HIT software (version 4.6.6; similarity threshold: 95%, coverage threshold: 90%). The Unigenes were separately for species and functional annotation. Abundancy statistics and differential comparative analysis were performed on species classification, functional annotation, and Unigene level, and KEGG database enrichment analysis was performed for differential Unigenes.

### 2.6 Statistical analysis

The normality of the data was assessed using the Shapiro-Wilk test and Histograms test, and non-normal distributions were either square-root or logarithmically transformed. Data that followed a normal distribution were analysed by one-way analysis of variance (ANOVA) with Duncan’s multiple range test (*P* < 0.05) using SPSS 23.0 software (IBM Corp., Armonk, NY,USA). Bioinformatic analysis of metagenomic sequencing data was performed using the OmicStudio tools at https://www.omicstudio.cn/tool. Mothur (https://www.mothur.org/) and Qiime (http://qiime.org/) software were used to plot the species accumulation curves and dilution curves, and calculate the α-diversity and β-diversity of bacterial and fungal. Python2 software was used to draw principal coordinate analysis charts (PCoA), non-metric multidimensional scale analysis charts (NMDS), and species abundance charts. Distance heat maps, enrichment bar charts, enrichment scatter plots, and Venn maps were drawn based on the OmicStudio platform (https://www.omicstudio.cn/tool). Compared differentially expressed genes (DEGs) with KEGG databases using R software and annotated their functions. The nonparametric factorial Kruskal-Wallis sum-rank test and linear discriminant analysis (LDA> 3.0) were used to identify biomarkers in different treatments, and graphically drawn using the LEfSe tool at https://www.omicstudio.cn/tool. Co-occurrence network analysis at the genus level was performed using the correlation network graph tool at https://www.omicstudio.cn/tool, and Gephi software (https://gephi.org/) was used to visualize network relationships. Redundancy analysis (RDA) was performed using Canoco5 software (Microcomputer Power, Ithaca, USA). The relationships between microbial diversity and environmental factors were investigated using Spearman’s correlation analysis, and the correlation analysis heat maps were drawn by Chiplot software (https://www.chiplot.online). Pathway analysis was plotted using Spsspro software (https://www.spsspro.com).

## 3 Results

### 3.1 Effects of fertilization on sorghum yield and soil physicochemical properties

The pH of unfertilized soil was 8.92 (**Table 1**). Both single application of chemical fertilizers and combined application of organic and inorganic fertilizers effectively reduced the soil pH (by 4.41%-7.89%), and the greater the proportion of organic fertilizer substitution, the greater the decreased in soil pH. The trend of BD changes was similar to that of pH. In addition, fertilization can effectively increase soil SWC (by 1.39%-16.54%). Short-term organic substitution had significant effects on soil nutrients. The SOC content of different treatments showed the order NF100>NF75>NF50>NF25>CF>CK. Compared with CF, NF25, NF50, NF75, and NF100 increased SOC content by 13.64%, 24.25% (*P* < 0.05), 32.75% (*P* < 0.05), and 54.62% (*P* < 0.05), respectively. The content of TN, TP, TK, AN and AK in soil increased first and then decreased with the increase of organic substitution ratio, and reached the maximum under NF50 treatment or NF75 treatment. Soil AP content increased with the increase of organic fertilizer replacement ratio, which was significantly higher than the CK and CF treatments (*P* < 0.05). Compared with CF treatment, NF50 treatment effectively increased the content of TN, TP, TK, AN and AP by 12.26%, 9.59%, 7.24%, 46.39% and 33.12%, respectively (*P* < 0.05). Similarly, organic fertilizer treatments significantly increased the activities of ALP, BG, LAP, and NAG. ALP activity gradually increased with the increase of organic substitution ratio, and significantly increased by 16.84%-27.38% compared with CF treatment (*P* < 0.05). The activities of BG, LAP and NAG increased first and then decreased with the increase of organic fertilizer substitution ratio, and reached the maximum under high ratio of organic fertilizer substitution (NF75), which were significantly increased by 20.63%, 84.85% and 64.30%, respectively, compared with CF treatment (*P* < 0.05).

**Table 1:** Effects of different fertilization treatments on soil physicochemical properties.

Soil properties	CK	CF	NF25	NF50	NF75	NF100
pH	8.92 ± 0.13a	8.52 ± 0.03b	8.27 ± 0.01c	8.26 ± 0.02c	8.25 ± 0.01c	8.21 ± 0.02c
Soil bulk density (BD, g/cm^3^)	1.54 ± 0.04a	1.52 ± 0.01ab	1.49 ± 0.02b	1.43 ± 0.04c	1.38 ± 0.01d	1.36 ± 0.01d
Soil water content (SWC, %)	7.83 ± 0.62c	8.35 ± 0.06bc	9.12 ± 0.12a	8.92 ± 0.17ab	8.36 ± 0.41bc	7.94 ± 0.19c
Electric conductivity (EC, μS/cm)	92.10 ± 1.00a	83.47 ± 7.87ab	79.37 ± 6.85b	83.93 ± 3.95ab	86.90 ± 2.84ab	87.63 ± 1.57ab
Soil organic carbon (SOC, g/kg)	8.56 ± 0.26e	9.49 ± 1.37de	10.78 ± 1.00cd	11.79 ± 1.13bc	12.60 ± 0.29b	14.67 ± 0.66a
Total nitrogen (TN, g/kg)	0.89 ± 0.04c	1.06 ± 0.03b	1.01 ± 0.07bc	1.19 ± 0.02a	1.05 ± 0.13b	0.94 ± 0.03bc
Total phosphorus (TP, g/kg)	1.56 ± 0.05b	1.67 ± 0.05b	1.68 ± 0.01b	1.83 ± 0.02a	1.90 ± 0.16a	1.56 ± 0.09b
Total potassium (TK, g/kg)	24.93 ± 0.67c	25.70 ± 0.32bc	26.22 ± 0.85b	27.56 ± 0.33a	26.17 ± 0.65b	25.33 ± 0.88bc
Available N (AN, mg/kg)	38.54 ± 3.42b	41.45 ± 4.85b	59.51 ± 1.36a	60.68 ± 4.61a	59.93 ± 4.83a	59.31 ± 7.06a
Available P (AP, mg/kg)	14.26 ± 0.69e	21.65 ± 2.98d	28.07 ± 0.67c	28.82 ± 0.46c	35.85 ± 1.26b	52.12 ± 1.62a
Available K (AK, mg/kg)	260.75 ± 3.04c	287.56 ± 2.05a	275.08 ± 3.11b	286.69 ± 2.44a	286.29 ± 6.07a	282.18 ± 3.91ab
β-D-Glucosidase (BG, U/g)	11.56 ± 0.15e	14.39 ± 0.32c	13.03 ± 0.43d	15.34 ± 0.58b	17.36 ± 0.55a	14.83 ± 0.39bc
Alkaline phosphatase (ALP, mg/g·24h)	0.19 ± 0.00e	0.20 ± 0.01d	0.22 ± 0.00c	0.23 ± 0.01b	0.24 ± 0.00b	0.25 ± 0.01a
L-Leucine aminopeptidase (LAP, U/g)	1.24 ± 0.03e	1.32 ± 0.06d	1.36 ± 0.03cd	1.42 ± 0.02b	2.44 ± 0.05a	1.39 ± 0.04bc
β-N-Acetylglucosaminidase (NAG, U/g)	6.50 ± 0.02d	6.19 ± 0.07d	6.48 ± 0.37d	9.02 ± 0.34b	10.17 ± 1.10a	7.51 ± 0.73c

Values represent mean ± SEM (n = 3). CK, Non-fertilization control; CF, 100% chemical fertilizer; NF25, NF50 and NF75 are 25%, 50% and 75% substitution of chemical fertilizer by organic fertilizer, respectively; NF100, 100% organic fertilizer. Different small letters represent significant difference among treatments (*P* < 0.05). The same below.

**Figure 1:**
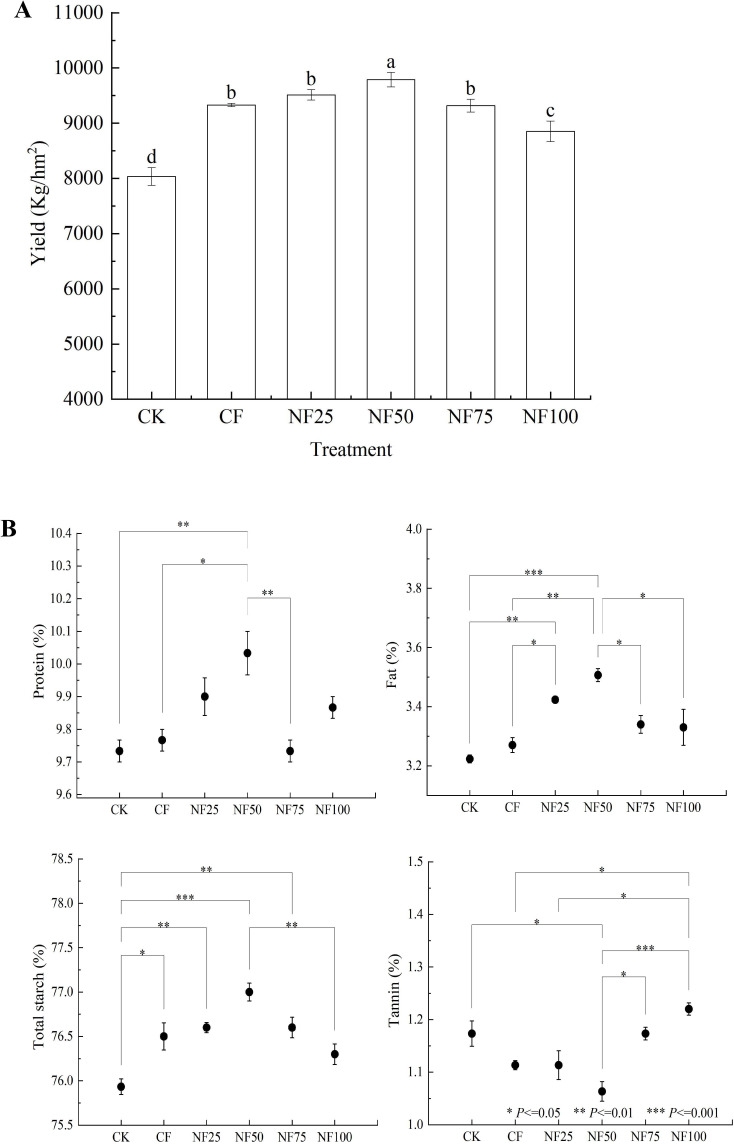
Effects of different fertilization treatments on sorghum grain yield **(A)** and quality **(B)**. Values represent mean ± SEM (n = 3). CK, Non-fertilization control; CF, 100% chemical fertilizer; NF25, NF50 and NF75 are 25%, 50% and 75% substitution of chemical fertilizer by organic fertilizer, respectively; NF100, 100% organic fertilizer. Different small letters represent significant difference among treatments (*P* < 0.05). Asterisks indicate significantly different values: * *P*<=0.05, ** *P*<=0.01, *** *P*<=0.001).

The sorghum yield was highest in the NF50 treatment (9789.9 kg/hm^2^), followed by the NF25, CF, NF75, NF100, and CK treatments (**Figure 1**). The sorghum yield after CF treatment was 9328.9 kg/hm^2^, and the yield from the plots that received the NF25 and NF50 treatments had higher, with significant differences between NF50 treatment and CF treatment (*P* < 0.05). In addition, organic fertilizer has a positive effect on sorghum grain quality. The content of protein, fat and total starch in sorghum grains treated with NF50 were the highest among all the treatments. Compared with CF treatment, the protein and fat contents of sorghum grains under the NF50 treatment increased by 2.73% and 7.24% (*P* < 0.05), respectively, along with a modest, insignificant increased in total starch content (by 0.65%), while the tannin content decreased by 4.49% (*P* < 0.05).

### 3.2 Effects of fertilization on soil microbial diversity

**Figure 2:**
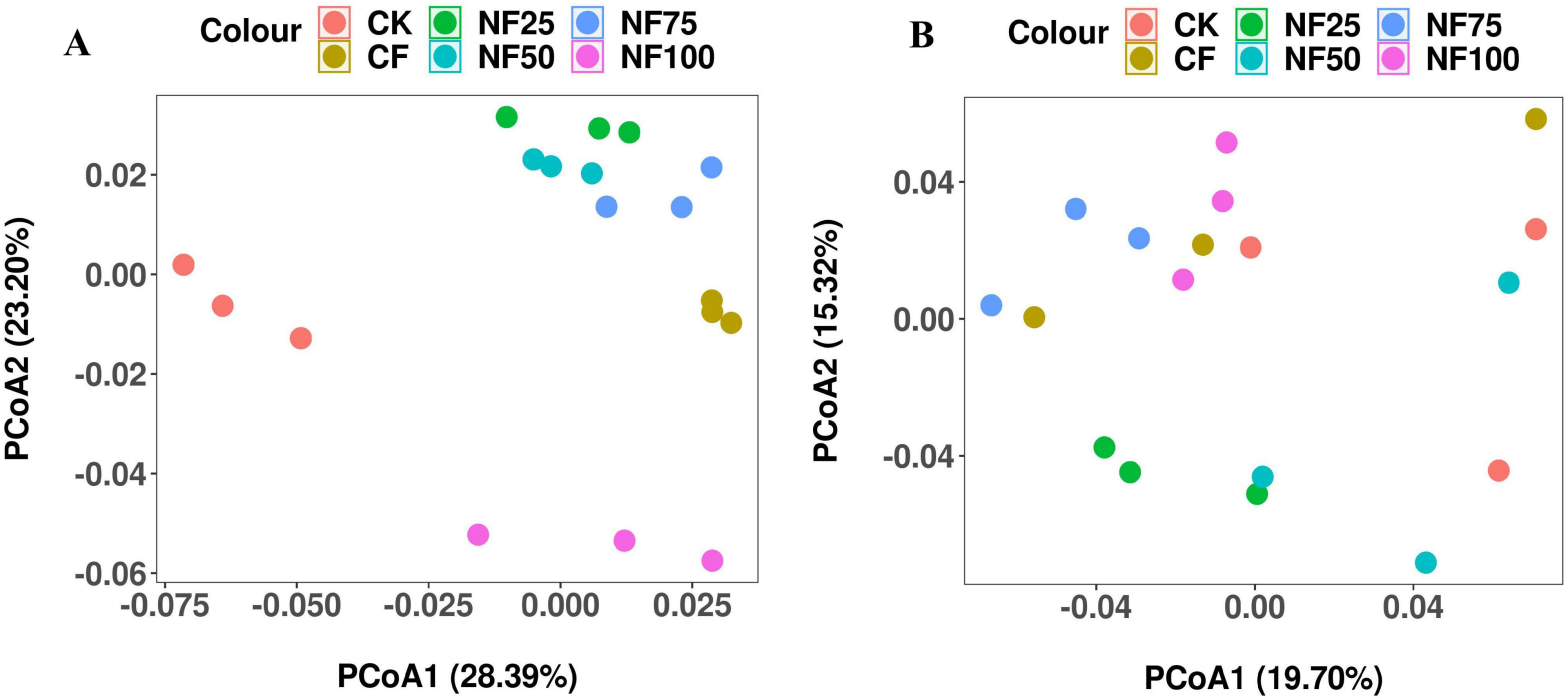
Effects of different fertilization treatments on soil microbial β diversity. Principal coordinate analysis (PCoA) plot of Bray-Curtis distance of bacterial **(A)** and fungal **(B)**.

Different fertilization treatments had a significant effects on soil bacterial α diversity, but not on fungal α diversity (**Table 2**). Compared with CK treatment, CF, NF25, and NF50 treatments significantly increased the observed species and Chao1 index (*P* < 0.05), with NF25 was the most significant; the Shannon index was significantly higher than the control only under NF75 treatment (*P* < 0.05). To evaluate the differences in bacterial and fungal β diversity, principal coordinate analysis (PCoA) was performed (**Figure 2**). The first two principal coordinates of the PCoA explained 51.59% and 35.02% of the variation in bacterial and fungal β diversity, respectively (**Figure 2**). The Unigenes in no fertilization (CK), pure chemical fertilizer (CF), organic fertilizer substitution(NF25, NF50, NF75) and pure organic fertilizer (NF100) treatments were separated along PCo1 and PCo2 (Adonis test, *P* = 0.001), indicating that organic fertilizer substitution measures had significant effects on bacterial community.

**Table 2:** Effects of different fertilization treatments on soil microbial α diversity.

Treatment	Bacteria			Fungi		
	Observed species	Shannon index	Chao1 index	Observed species	Shannon index	Chao1 index
CK	12919.33 ± 93.98c	8.52 ± 0.01b	13081.0 ± 147.05b	137.00 ± 1.00a	4.96 ± 0.18ab	140.62 ± 1.41a
CF	13024.33 ± 33.50ab	8.56 ± 0.05ab	13220.90 ± 38.52a	136.67 ± 4.93a	4.90 ± 0.05ab	143.23 ± 6.02a
NF25	13083.00 ± 42.33a	8.51 ± 0.02b	13300.78 ± 44.50a	137.33 ± 3.51a	4.93 ± 0.06ab	144.95 ± 4.79a
NF50	13005.67 ± 43.88ab	8.44 ± 0.04c	13242.02 ± 60.94a	135.33 ± 3.06a	5.03 ± 0.08a	137.77 ± 3.35a
NF75	12959.00 ± 10.58bc	8.57 ± 0.02a	13073.00 ± 3.10b	135.33 ± 3.21a	4.80 ± 0.06b	138.44 ± 4.31a
NF100	12922.00 ± 56.00c	8.35 ± 0.03d	13100.89 ± 111.13b	135.67 ± 1.53a	4.94 ± 0.06ab	138.91 ± 1.20a

Different small letters represent significant difference among treatments (*P* < 0.05).

### 3.3 Effects of fertilization on soil microbial community composition

**Figure 3:**
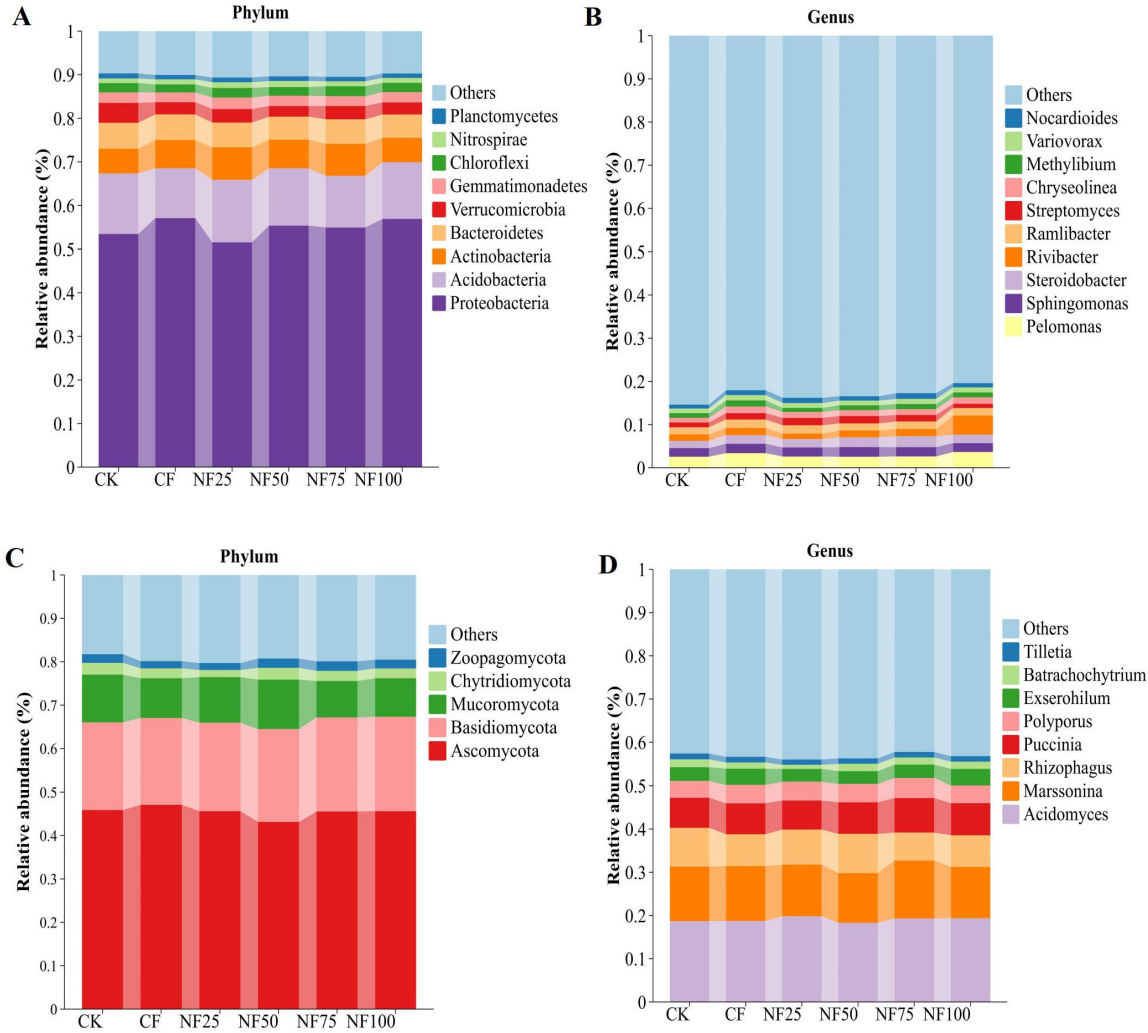
Effects of different fertilization treatments on microbial community composition. **(A)** Dominant bacteria (relative abundance> 1%) at the phylum level; **(B)** dominant bacteria (top 10 in relative abundance) at the genus level; **(C)** dominant fungal (relative abundance> 1%) at the phylum level; **(D)** dominant fungal (relative abundance> 1%) at the genus level.

The bacterial sequences were distributed in 134 phyla 2087 genera. The bacterial communities of all soil samples were mainly composed of Proteobacteria (54.87%), Acidobacteria (12.96%), Actinomycetes (6.52%), Bacteroidetes (5.63%) and Verrucobacteria (3.12%), accounting for more than 80% of the relative abundances of all phyla (**Figure 3A**). Other dominant bacterial phyla (relative abundance > 1%) included Gemmatimonadetes (2.38%), Chloroflexi (2.11%), Nitrospirae (1.19%), and Planctomycetes (1.06%). Compared with the control group, the relative abundances of Proteobacteria, Actinobacteria, and Nitrospirae were notably enhanced in the CF and NF50 treatments; conversely, the relative abundances of Acidobacteria, Verrucomicrobia and Planctomycetes markedly decreased (*P* < 0.05; **Supplementary Table S1.1**). The top 10 genera of bacterial in all soil samples were *Pelomonas* (5.76%), *Sphingomonas* (4.24%), *Steroidobacter* (4.14%), *Rivibacter* (4.03%), *Ramlibacter* (3.55%), *Streptomyces* (2.81%), *Chryseolinea* (2.73%), *Methylibium* (2.31%), *Variovorax* (2.29%) and *Nocardioides* (2.20%), which accounted for less than 20% of the relative abundances of all genera (**Figure 3B**). These dominant bacterial genera (top 10 genera) belong to four phyla (Proteobacteria, Actinobacteria, Bacteroidetes and Nitrospirae). Compared with CK treatment, a high ratio of organic fertilizer substitution (NF75) treatment had the greatest impact on the relative abundance of dominant bacterial genera, and 7 of the top 10 bacterial genera were significantly increased (*P* < 0.05; **Supplementary Table S1.2**).

A total of 7 phyla and 126 genera in the fungal community were detected in all samples. The dominant fungal phyla (relative abundance > 1%) were Ascomycota (45.48%), Basidiomycota (20.86%), Mucoromycota (9.90%), Chytridiomycota (2.32%), and Zoopagomycota (1.94%). (**Figure 3C**). Application of organic fertilizer increased the relative abundance of Basidiomycota compared to CF treatment, but the difference did not reach a significant level (**Supplementary Table S1.1**). Moreover, the dominant fungal genera (relative abundance > 1%) across all samples were *Acidomyces, Marssonina, Rhizophagus, Puccinia, Polyporus, Exserohilum, Batrachochytrium* and *Tilletia*, accounting for 55.34% of all sequences (**Figure 3D**). Fertilization treatments had no significant effect on the dominant fungal genera (**Supplementary Table S1.2**).

Biomarkers with significant differences in abundance between pure chemical fertilizer treatment and organic fertilizer substitution treatments were identified using the nonparametric factorial Kruskal-Wallis sum-rank test and linear discriminant analysis (LDA> 3.0). Covering the bacterial phylum to species level, the number of biomarkers with significant differences in abundance between NF25 and CF treatments, NF50 and CF treatments, and NF75 and CF treatments were 62, 49, and 32, respectively. Among these, 29 shared biomarkers were found. 11 biomarkers were significantly enriched in both low and medium ratio of organic fertilizer substitution treatment; 12 biomarkers were significantly enriched in both low and high ratio of organic fertilizer substitution treatments. The phyla represented by the biomarkers enriched in the the organic fertilizer substitution treatments were classified as Actinobacteria, Acidobacteria and Chloroflexi. 6 biomarkers were significantly enriched in the CF treatment and classified as Proteobacteria. In addition, NF25, NF50, and NF75 treatments significantly enriched 12, 15, and 8 unique biomarkers, respectively (**Supplementary Table S2.1**). Covering the fungal phylum to species level, The biomarkers with significant differences between the NPK treatment and the organic fertilizer subtitution treatments were 6. Among these, 1 biomarker (*Hyaloscyphaceae* (family) was significantly enriched in all the organic fertilizer substitution treatments, which belong to Ascomycota; 5 biomarkers were significantly enriched in CF treatment and all belong to Mucoromycota. (**Supplementary Table S2.2**).

### 3.4 Impacts of fertilization on the co-occurrence network

**Figure 4:**
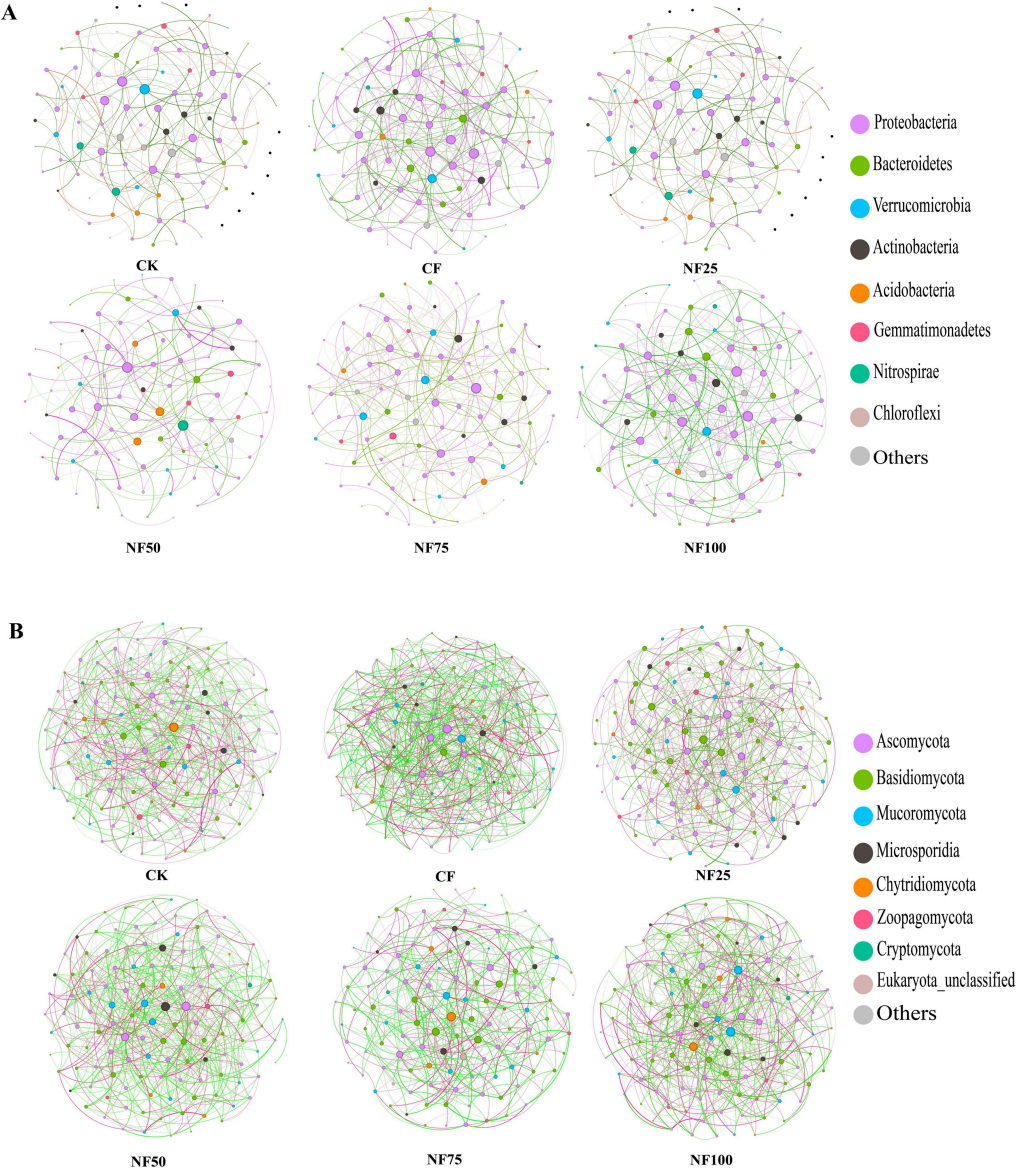
The co-occurrence network analysis of bacteria **(A)** and fungi **(B)** at the genus level. Nodes (colored dots) represent the genera involved in the networks, the different colored dots represent the different phyla to which the genera belong. The red line represents the positive correlation, and the green line represents the negative correlation.

Co-occurrence network analysis was conducted at the genus level to explore the effects of chemical fertilizer reduction combined with organic fertilizer on microbial correlations (**Figures 4A, B**). In the bacterial network (**Supplementary Table S3**), inorganic fertilization (CF) resulted in the highest number of nodes (92), number of edges (256), network density (0.061) and average degree(5.565), and the lowest average path length (2.736) and modularity (0.445). The organic fertilizer substitution treatments (NF25 and NF50) reduced the number of nodes, number of edges, network density, and average degree, while increased the average path length and modularity compared with CF treatment. The network density and the proportion of positive edges of NF50 treatment were 0.043 and 38.790%, respectively, which were the lowest among all fertilization treatments. The average path length (3.525)and modularity (0.559) of NF25 treatment were highest among all treatments. The biomarkers at the genus level with significant differences between the CF treatment and the organic fertilizer substitution treatments were located in the network. In the fungal network (**Supplementary Table S3**), the number of edges (604), network density (0.078), average degree (9.438), and average clustering coefficient (0.084) of inorganic fertilization (CF) were the highest, while the average path length (2.385) and modularity (0.315) were the lowest. The organic fertilizer substitution treatments decreased the number of edges, average degree, network density and average clustering coefficient, and increased the average path length and modularity compared with CF treatment. NF75 treatment resulted in the lowest network density (0.051) and highest modularity (0.400).

### 3.5 Effects of fertilization on soil functional characteristics

**Figure 5:**
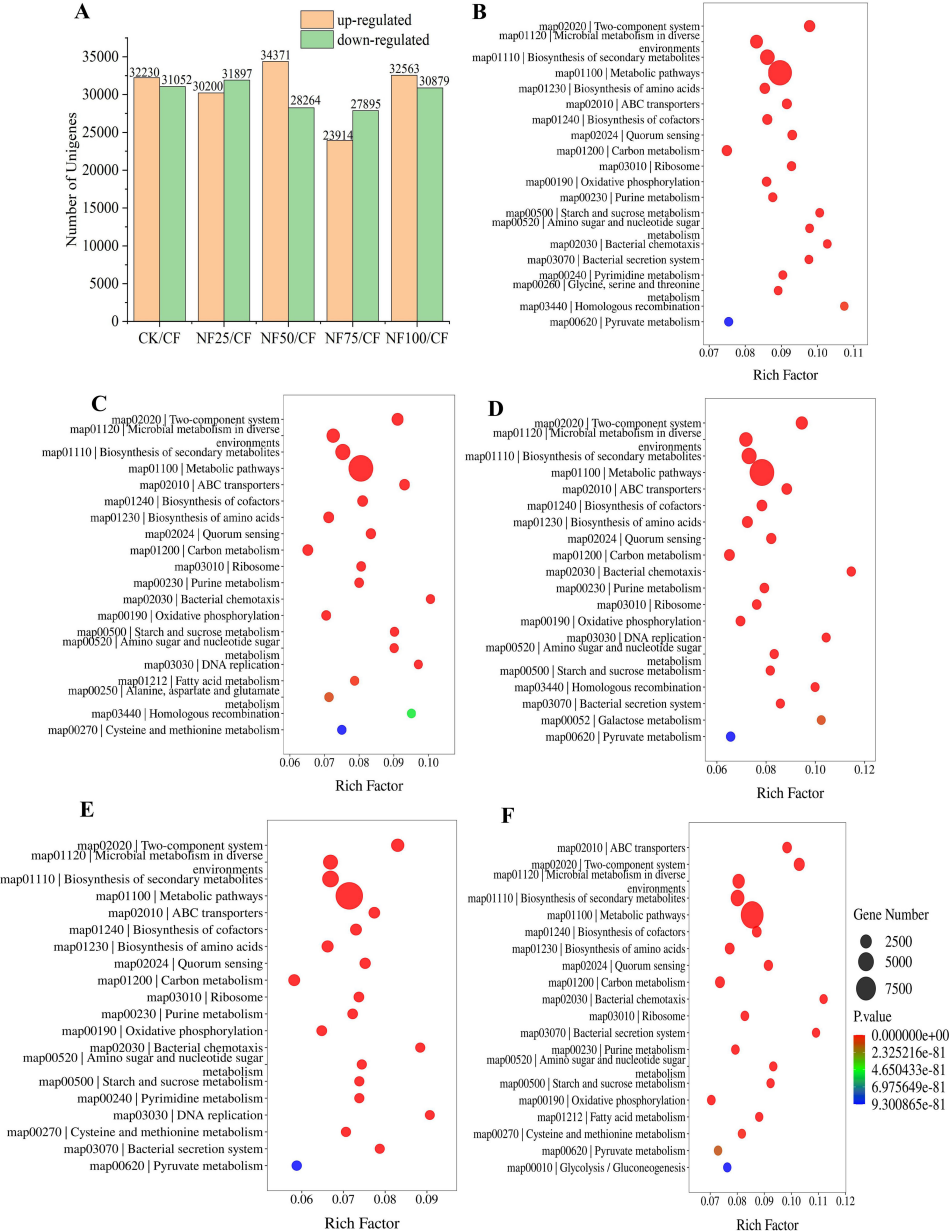
KEGG enrichment analysis of genes in sorghum rhizosphere soil under different fertilization treatments. **(A)** DEGs in different treatments. KEGG enrichment analysis of DEGs between CK **(B)**, NF25 **(C)**, NF50 **(D)**, NF75 **(E)**, NF100 **(F)** and CF. The size and color of the bubbles correspond to the gene number and the P value enriched in the pathway. The rich factor represents the ratio of the number of genes mapped to a certain pathway to the total numbers of genes mapped to this pathway.

The histogram in **Figure 5A** displayed that 62097, 62635, 51809, and 63442 DEGs were identified in NF25/CF, NF50/CF, NF75/CF, and NF100/CF, respectively; among which were 30200, 34371, 23914, and 32563 up-regulated and 31897, 28264, 27895, and 30897 down-regulated, respectively.

Based on KEGG enrichment analysis, we found 28139, 19777, 23662, 17956, and 26439 DEGs from CK/CF, NF25/CF, NF50/CF, NF75/CF, and NF100/CF, respectively, and these genes were significantly enriched in the 160,157,156,159, and 158 KEGG pathways, respectively (**Supplementary Table S4.1**). Among the pathways of significant enrichment, the top two pathways with the highest numbers of DEGs were “Metabolic pathways (map01100)” and “Biosynthesis of secondary metabolites (map01110)” (**Figures 5B–F**). A total of 9691, 10967, 10016, 9969 and 1076928 DEGs were associated with the pathway of “Metabolic pathways (map01100)” in CF/CK,NF25/CK, NF50/CK, NF75/CK and NF100/CK, respectively. This result indicates that this pathway is more active in sorghum rhizosphere soil under organic fertilizer substitution than that under pure chemical fertilizer. The number of DEGs associated with NF25/CF, NF50/CF, NF75/CF and NF100/CF were 8699, 8465, 7722 and 9244, respectively, it shows that this pathway is most active in pure organic fertilizer treatment (NF100), followed by NF25, NF50, and NF75 (**Supplementary Table S4.2**). The impact of organic fertilizer substitution on the “Carbon metabolism(map01200)” pathway was similar to that of “Metabolic pathways (map01100)”. KEGG analysis also identified that 4060, 4653, 4339,4184 and 4510 DEGs were involved in the pathway of “Biosynthesis of secondary metabolites (map01110)” in CF/CK, NF25/CK, NF50/CK, NF75/CK and NF100/CK groups, respectively. This result indicates that the application of organic fertilizers enhanced secondary metabolite biosynthesis in sorghum rhizosphere soil.

### 3.6 Impacts of fertilization on the correlations between environmental factors and microorganisms

**Figure 6:**
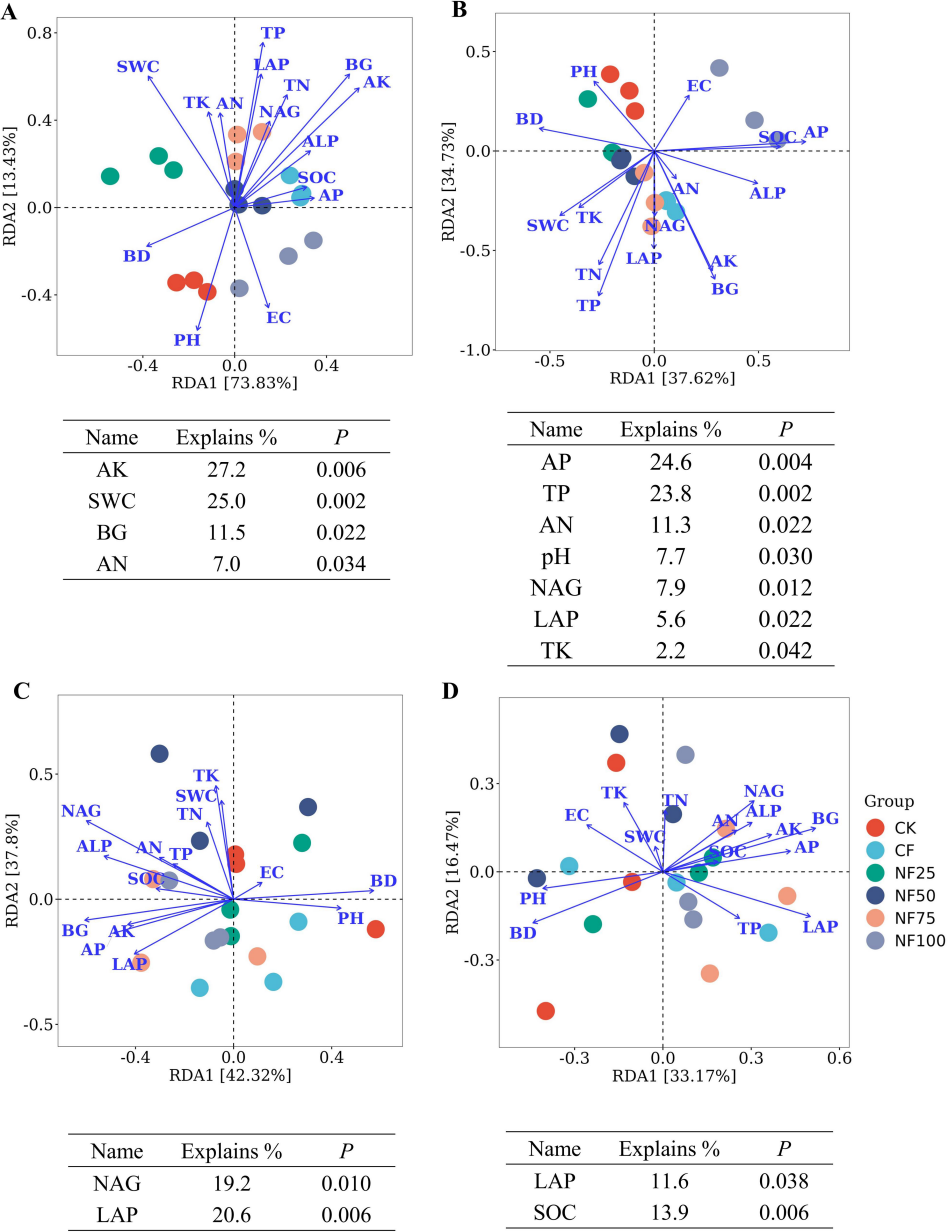
Redundancy analysis (RDA) between community structure of **(A)** all bacterial phyla, **(B)** all bacterial genera, **(C)** all fungal phyla and **(D)** all fungal genera with soil physicochemical factors. The statistically significant physicochemical factors and their explanations are shown below the plots.

The RDA revealed the influences of environmental factors on microbial community structures of sorghum rhizosphere soil (**Figure 6**). The first axis of the RDA explained 73.83% of the variation in bacterial community, and the second axis explained 13.43% of the variation. AK (explanation rate = 27.2%) had the greatest impact on bacterial community structure, followed by SWC, BG, and AN. All samples were distributed across 7 fungal phyla. The first two axes of RDA accounted for 80.12% of the total variation in fungal community structure (PC1 = 42.32%, PC2 = 37.80%). NAG and LAP significantly impacted the fungal community structure and explained 19.2% and 20.6% of the variation, respectively (*P* < 0.05). At the genus level, RDA of the effects of soil physicochemical properties on the community structures of bacterial and fungal showed that AP, TP, AN, pH, NAG, LAP and TK had significant effects on the bacterial community structure (*P* < 0.05), and LAP and SOC were the two environmental factors that significantly affected the fungal community structure (*P* < 0.05). The RDA results indicate that AN and LAP were important factors influencing the communities of soil bacterial and fungal, respectively.

**Figure 7:**
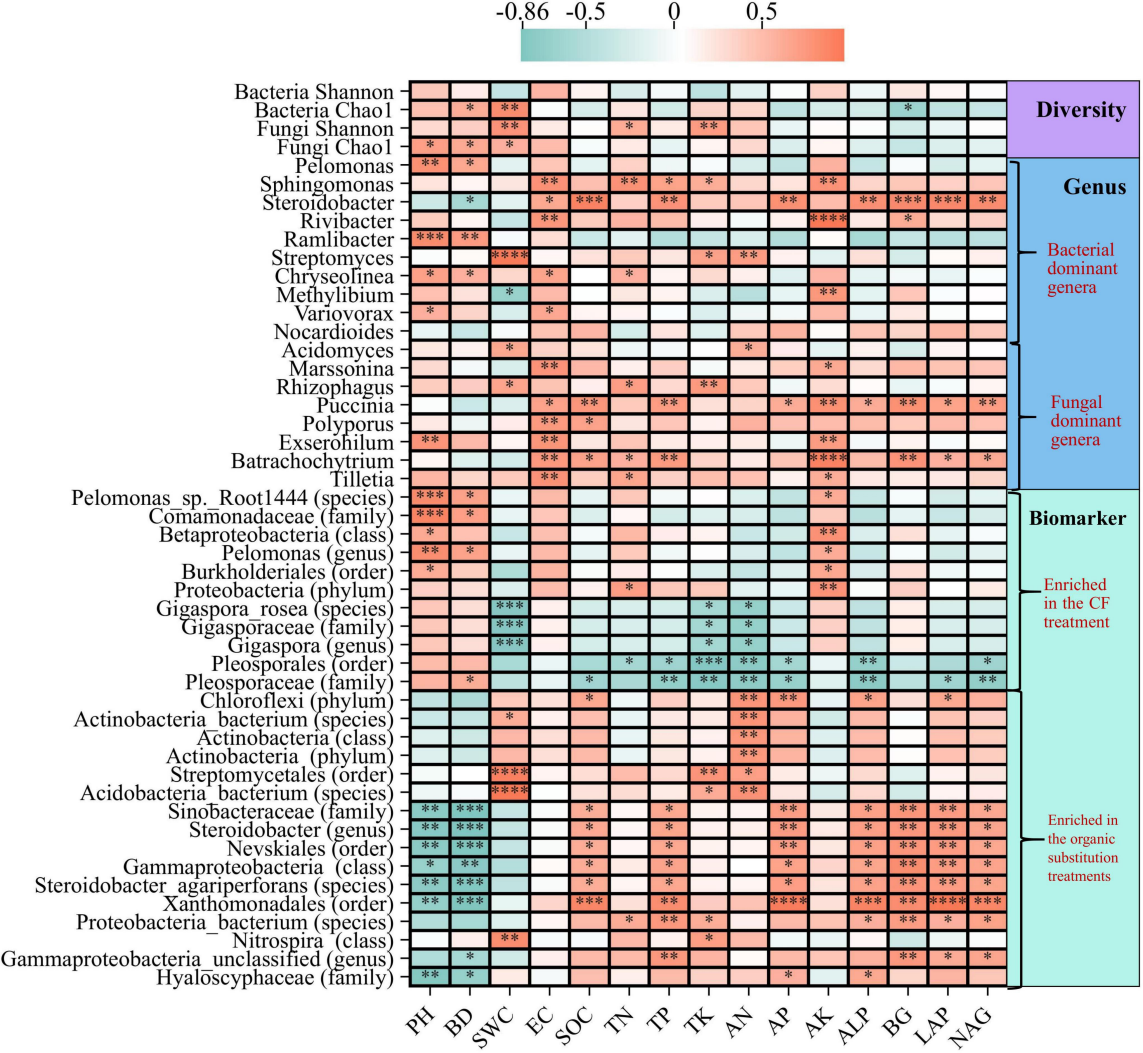
Spearman correlation analysis heat maps of environmental factors and microbial diversity, dominant genera, and biomarkers. Red indicates a positive correlation, and blue indicates a negative correlation (*P<0.05; **P<0.01; ***P<0.001; ****P <0.001).

Spearman correlation analysis were used to further characterize the relationships among bacterial diversity, fungal diversity, dominant phyla, dominant genera, biomarkers, and environmental factors (**Figure 7**; **Supplementary Figure S1**). SWC was significantly positively correlated with bacterial Chao1 index and fungal Shannon index (*P* < 0.01). The Spearman correlations between dominant phyla and environmental factors showed that the dominant bacterial phyla Actinobacteria and Nitrospirae were significantly positively correlated with SWC, TN, TP and TK, while Acidobacteria and Planctomycetes were significantly negatively correlated with AK; the dominant fungal phylum Basidiomycota was significantly positively correlated with SOC, TK, AN, AP, BG, LAP and NAG (*P* < 0.05, *P* < 0.01, or *P* < 0.001). The Spearman correlations between dominant genera and environmental factors found that the dominant bacterial genus *Sphingomonas* was significantly positively correlated with TN, TP, TK and AK, *Steroidobacter* was significantly positively correlated with SOC, TP, AP, ALP and BG, *Streptomyces* was significantly positively correlated with TK and AN (*P* < 0.05, *P* < 0.01, *P* < 0.001, or *P* < 0.0001); the fungal dominant genus *Rhizophagus* was significantly positively correlated with TN and TK (*P* < 0.05 or *P* < 0.01). In the CF treatment, the bacterial biomarkers were positively correlated with pH, BD, and AK, and most of correlations were significant (*P* < 0.05, *P* < 0.01, or *P* < 0.001); and the fungal biomarkers were significantly negatively correlated with most environmental factors except pH, BD and AK (*P* < 0.05, *P* < 0.01, or *P* < 0.001). However, the biomarkers enriched in organic substitution treatments showed completely opposite correlations, which were positively correlated with most environmental factors except pH, BD and AK. In summary, the biomarkers enriched in CF treatment and organic substitution treatments showed opposite trends for the same environmental factors, and pH, BD and AK showed opposite correlation trends with other environmental factors.

**Figure 8:**
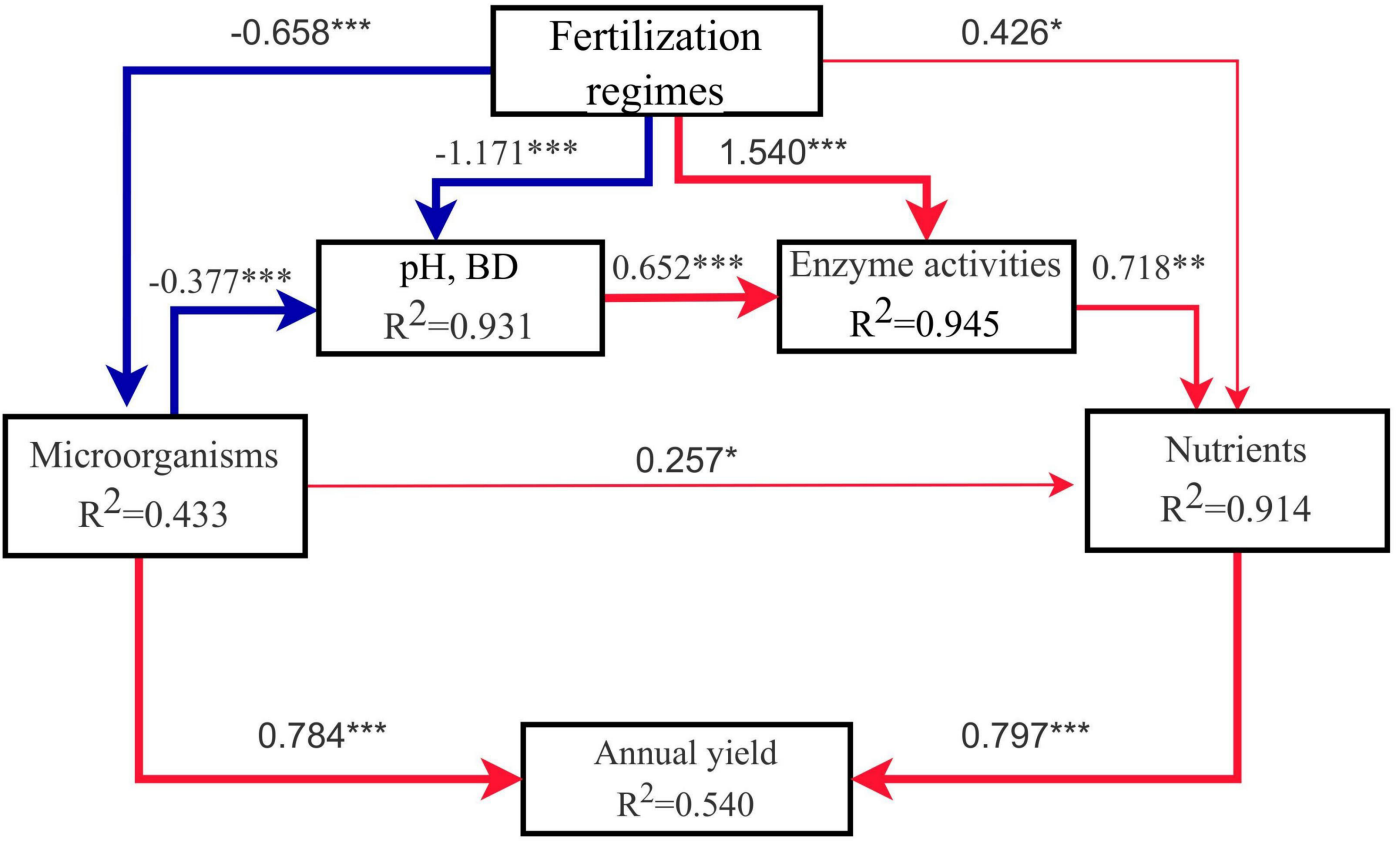
Path analysis plots based on the linear regression approach. The thicknesses of the arrow reflects the significance of the path (*P<0.05; **P < 0.01; ***P < 0.001), the red line represents the positive correlation, and the blue line represents the negative correlation. The model goodness of fit index (GFI) was 0.941.

Pathway analysis was used to analyse the effects of organic substitution treatments on soil microorganisms, soil pH, soil nutrients, soil enzyme activities, and sorghum annual yield. The model fit the data well (χ^2^ = 7.388, *P* = 0.193, GFI = 0.941, SRMR = 0.040), explained 43.3% of the variation in soil microorganisms, 93.1% of the variation in soil pH and BD, 91.4% of the variation in soil nutrients, 94.5% of the variation in soil enzyme activities, and 54.0% of the variation in sorghum yield (**Figure 8**). The results showed that the fertilization regimes significantly affected the soil pH and BD (λ = -1.171, *P* < 0.001), soil nutrients (λ = 0.426, *P* < 0.05), soil enzyme activities (λ = 1.540, *P* < 0.001), and microbial community diversity (λ = -0.658, *P* < 0.001). Soil nutrient status directly affected the sorghum yield by changing nutrient supply (λ = 0.797, *P* < 0.001). The impact of microorganisms on sorghum yield can be directly influenced by changing microbial community diversity (λ = 0.784, *P* < 0.001), or indirectly mediated by pH and BD (λ = -0.377, *P* < 0.001), enzyme activities (λ = -0.652, *P* < 0.001) and soil nutrients (λ = 0.718, *P* < 0.01).

## 4 Discussion

### 4.1 Short-term responses of soil properties and yield to chemical fertilizer reduction combined with organic fertilizer

The chemical fertilizer reduction combined with organic fertilizer has a positive impact on crop productivity and sustainable development. However, the impacts of the short-term chemical fertilizer reduction combined with different ratios of organic fertilizer on sorghum yield are still unclear. This study applied organic fertilizer for the first time in soil treated with chemical fertilizer alone for 5 consecutive years, and explored the effects of short-term organic fertilizer substitution on sorghum yield, quality, soil physicochemical properties, microbial community structure and functions.

Soil fertility is affected by factors such as soil nutrients, soil physical characteristics and soil water content, which directly affects plant productivity (Kooch et al., 2024). Liu et al. (2021a) found that long-term organic fertilizer substitution increased soil organic matter content and nutrients levels, and reduces soil acidification, and these changes collectively promoted the improvement of rice yield, this observation was confirmed by our findings. The fertilizer reduction combined with cow manure organic fertilizer significantly increased soil organic matter content (**Table 1**). This may be due to the fact that cow manure itself is rich in organic carbon, and the input of exogenous carbon can effectively increase the content of soil organic matter by stimulating microbial mineralization (Wu et al., 2022). The increase of organic matter content provides abundant nutrients for microbial growth and reproduction, enhancing the activation of soil enzymes and the supply of nutrients (Guo et al., 2023). Under the medium ratio of organic fertilizer substitution (NF50) treatment, soil TN, TP, AN, AP, AK, ALP, BG, LAP, and NAG were significantly improved, and the yield was significantly increased (**Table 1**; **Figure 1A**). The combined application of organic and inorganic fertilizers can slow down the release and loss of nutrients, improving the efficiency of nutrient utilization while increasing crop yield and quality (Guo et al., 2023, 2024b). The results of path analysis further confirmed that changes in soil nutrients under different treatment conditions were the factor that explained the highest proportion of changes in sorghum annual yield (**Figure 8**). Compared with the application of traditional inorganic fertilizers, the application of organic fertilizers also has a positive impact on the nutrient composition of vegetables and fruits (Akram et al., 2024; Sahu and Joseph, 2024). Our research obtained similar conclusions, organic fertilizer substitution significantly increased the protein, fat and total starch contents of sorghum grains, while significantly reducing the tannin content of sorghum grains (**Figure 1B**). In addition, the application of organic fertilizers can optimize soil aggregate structure by increasing organic matter, thereby improving soil physical properties, and increasing soil water retention (Ma et al., 2022). Our study found that appropriate organic fertilizer application could reduce soil pH and BD, increase SWC (**Table 1**). The previous conclusions regarding the effects of the combined application of organic and inorganic fertilizers on soil pH were inconsistent. This may be due to differences in soil acidity and alkalinity, Long-term application of high amount of manure in acidic soil can significantly increase soil pH and effectively alleviate soil acidification, while long-term application of high amount of manure in alkaline soil can effectively reduce soil pH (Guo et al., 2024a). The above results indicate that organic fertilizer substitution can comprehensively improve the soil environment in terms of soil physical properties, total nutrients, available nutrients, and enzyme activities, and promote the improvement of sorghum yield and quality.

### 4.2 Short-term responses of microbial community composition and function to the substitution of organic manure for chemical fertilizer

The increase of soil microbial diversity is closely related to the improvement of soil ecosystem functions. It is generally believed that the higher the soil microbial diversity, the stronger the ability to resist external environmental interference, and the more stable the soil ecosystem (Wang et al., 2024a, b). The results reported herein demonstrate that organic-manure substitution treatments significantly increased the distribution of bacterial diversity in sorghum rhizosphere soil, but had no significant effect on fungal diversity (**Table 2**; **Figure 2**). This observation is consistent with the reports of Ren et al. (2021) indicating that fungal community was less sensitive to organic fertilizer substitution than bacterial community. This may be due to the higher N demand and sensitivity of bacteria, while fungal diversity was mainly closely related to crop type conversion, so future studies should consider the influence of crop type conversion (Wang et al., 2024a).

The adjustment of fertilization pattern resulted in a drastic change in bacterial community composition. In this study, the dominant bacterial phyla, such as Proteobacteria, Actinobacteria, and Nitrospirae notably increased in the NF50 and NF75 treatments; conversely, Aciobacteria and Planctomycetes were less abundant (**Supplementary Table S1.1**). Proteobacteria and Actinobacteria can degrade lignin and cellulose, and play an important role in soil carbon cycling. As eutrophic bacteria, they can multiply rapidly in the environment with abundant resources (Wang et al., 2021, 2024b; Hu et al., 2023). Thus, these bacteria require more soil nutrients than others, which further explains the increases in Actinobacteria and their close association with higher soil nutrients (TN, TP, TK) in the organic substitution treatments (**Supplementary Figure S1**). Similarly, the negative correlation between Planctomycetes and soil nutrients (SOC, AP, AK, ALP) confirmed that Planctomycetes belong to oligotrophic bacteria, which were generally considered to be suitable for growth and reproduction in oligotrophic environments which resource limited (Chen et al., 2021; Zha et al., 2024). It is known that Nitrospirae participate in nitrification, which are related to nitrogen cycle and geological mineralization, and play a key role in soil nitrogen metabolism (Liu et al., 2020). The relative abundance of soil Nitrospira in manure fertilization treatments was significantly higher than that in CK and CF treatments, and showed a significant positive correlation with TN and AN (**Supplementary Figure S1**), indicating that the increase of N availability in cow manure fertilization can enrich bacteria to participate in the nitrogen cycle. Moreover, organic fertilizer substitution significantly enriched biomarkers, such as *Streptomycetales* (order)、*Xanthomonadales* (order)、*Steroidobacter_agariperforans* (order) and *Nitrospira* (class), which were positively correlated with most physicochemical factors except AK (**Figure 7**; **Supplementary Table S2**). The order *Streptomyces* are reported to be an important plant growth-promotiong rhizosphere (PGPR) that can promote plant growth and reduce the occurrence of plant diseases through beneficial interactions with plant roots (Lin et al., 2019). The order *Xanthomonadales* are usually isolated from the rhizosphere of plants and are associated with reducing oxidative stress and promoting plant growth (Faist et al., 2023). The order *Steroidobacter_agariperforans* have biodegradation function (Gong et al., 2016). *Nitrospira* (class) play an important role in the nitrogen cycle by performing nitrite oxidation in the second step of nitrification (Liu et al., 2020). The enrichment of these biomarkers and beneficial microorganisms in organic fertilizer substitution treatments indicate that the addition of organic fertilizers create a more favorable environment for nutrient release and crop growth.

Among dominant fungi phyla, the relative abundance of Basidiomycota increased as the proportion of organic fertilizer was increased, and was mainly positively influenced by soil SOC, TK, AN and AP (**Supplementary Table S1.1**; **Supplementary Figure S1**), which confirmed that the increase of the relative abundance of Basidiomycota had a positive impact on the mineralization and nutrient release of soil exogenous organic matter (Guo et al., 2018). In addition, under medium ratio of organic fertilizer substitution treatment, the relative abundance of *Rhizophagus* (dominant fungi genera) was the largest, which was positively correlated with TN and TK (**Supplementary Table S1.2**; **Figure 7**). *Rhizophagus* belong to the phylum Glomeromycota, they form a symbiotic relationship with plants, known as Arbuscular Mycorrhizal (AM). this symbiotic relationship plays an important role in palnt nutrient absorption and resistance to environmental stress (He et al., 2023). Adequate nitrogen supply in soil can drive the auxiliary effect of AMF on plant stress resistance (Giambalvo et al., 2022). Moreover, we observed that *Hyaloscyphaceae* (family) were significantly enriched in organic fertilizer substitution treatments and positively correlated with all environmental factors except pH, BD, EC and AK (**Figure 7**; **Supplementary Table S2**). Luo et al. (2023a) showed that *Hyaloscyphaceae* had good phosphate-dissolving ability and can change rhizosphere soil conditions to promote the growth of host plants. The above results suggest that changes in fungal community composition under organic substitution may promote crop growth by enhancing nutrient mineralization and plant protection.

Fertilization measures have important effects on the structure and functional diversity of soil microbial communities. The network analysis provided insights into the effects of different fertilization methods on soil microecology (Liu et al., 2021a). In the current study, inorganic fertilization improved the network density and positive correlation density of bacteria, and also had a certain effect on the increase of fungal network density, while medium ratio of organic fertilizer substitution treatment reduced the network density and positive edge ratio of bacteria and fungi, which is consistent with the results of Fang et al. (2024) (**Figure 4**; **Supplementary Table S3**). The reduction of network density and positive correlation between genera caused by organic fertilizer substitution may be related to the balance and diversification of soil nutrients (Liu et al., 2021a). Some theoretical studies predict that networks composed of weak interactions are more stable than those composed of strong interactions, and that the presence of modularity and negative interactions in the network increase the stability of the network under disturbances (Coyte et al., 2015; de Vries et al., 2018; Stouffer and Bascompte, 2011). Under organic substitution, the greater number of negative interactions may contribute to enhance resistance to pathogens (Liu et al., 2021a). In addition, organic fertilizer substitution treatments improved the metabolic pathways, biosynthesis of secondary metabolites, and carbon metabolism (**Figure 5**). Metabolites of soil microorganisms can promote the dissolution of insoluble substances in soil, and some secondary metabolites have inhibitory effects on the reproduction of pathogenic microorganisms (Li et al., 2024; Song and Tian, 2024). Among the biomarkers enriched in the organic fertilizer treatments, Actinobacteria phylum (Yang et al., 2022). and Chloroflexi phylum (Xian et al., 2020) have the ability to hydrolyze and mineralize refractory organic carbon, which may be related to the improvement of carbon cycle function. In summary, organic substitution increased the diversity of bacterial community and the relative abundance of beneficial microorganisms, enhanced network stability of bacterial and fungal networks, and improved soil microbial metabolic cycle. The improvement of the structure and functions of microbial communities caused by organic fertilizers may act together to contribute to crop growth and yield increase by improving soil structure, promoting organic matter mineralization, inhibiting pathogen growth, and enhancing plant stress resistance.

### 4.3 Linkages between soil environmental factors and microbial community diversity and composition

The path analysis results of this study showed that short-term organic fertilizer substitution increased soil nutrients and directly contributed to the increase of sorghum yield. Organic fertilizer substitution increased soil microbial community diversity, providing a direct contribution to the increase in sorghum yield; and the improvement of microbial diversity can also indirectly improve sorghum yield through pH and BD, soil nutrients and enzyme activities (**Figure 8**). Microbial community structure is closely related to environmental factors. RDA results showed that AN and LAP were important factors affecting the changes of soil bacterial and fungal communities, respectively (**Figure 6**). Similar results were obtained in the studies of Luo et al. (2023b) and Xu et al. (2023). Moreover, correlation analysis showed that physicochemical factors such as SOC, TP, AP, and ALP were significantly associated with *Steroidobacter* (dominant genera) as well as with *Pleosporaceae* (family), *Nevskiales* (order), *Gammaproteobacteria* (calss) and *Xanthomonadales* (order), and AN was significantly correlated with biomarkers such as *Gigaspora_rosea* (species), *Pleosporales* (order), *Chloroflexi* (phylum), and *Actinobacteria* (phylum) (**Figure 7**). pH, BD, EC, and AK showed opposite correlation trends with other environmental factors. The biomarkers enriched in inorganic treatment and organic fertilizer replacement treatments showed opposite correlation trends for the same environmental factors. These results suggest that organic fertilizer substitution can comprehensively improve soil physicochemical properties, enzyme activities, and microbial community structure and functions, and their synergistic effect may jointly promote the increase of sorghum yield.

## 5 Conclusion

Short-term combined application of manure and chemical fertilizer effectively improved sorghum productivity and quality. Our results confirmed that sorghum yield was the highest under the medium ratio of organic fertilizer substitution. Organic fertilizer substitution reduced rhizospheric soil pH and BD significantly, thereby significantly improving soil enzyme activities (BG, ALP, LAP, and NAG), and soil nutrients (SOC, TN, TP, TK, AN, and AP), compared to the pure chemical fertilizer treatment. Organic fertilizer application also has positive effects on the increase of protein, fat, and total starch contents in sorghum grains, as well as the decrease of tannin content. Soil bacterial diversity changed more than fungal diversity owing to organic fertilizer substitution at the levels tested herein. The dominant bacterial phyla Proteobacteria and Actinobacteria significantly increased and Acidobacteria and Planctomycetes significantly decreased in the medium ratio of organic substitution treatment. However, the dominant fungal phyla did not respond significantly to organic substitution. Additionally, an increase in modularity and a decrease in network density of bacterial and fungal co-occurrence networks were observed in the organic fertilizer substitution treatments. The addition of organic fertilizers also improved soil microbial metabolic pathways, biosynthesis of secondary metabolites, and carbon metabolism. Furthermore, the variations in AN and LAP were considered to be the main driving parameters of soil chemical properties with a high potential for controlling soil microbial diversity. The changes of soil nutrients and microbial communities caused by organic fertilizers jointly promoted the increase of sorghum yield. Our research provides theoretical support for rational utilization of cow manure fertilization in sorghum fields, and is great significance for protecting soil ecological environment and promoting sustainable agricultural development.

## Data Availability

The datasets presented in this study can be found in online repositories. The names of the repository/repositories and accession number(s) can be found below: https://www.ncbi.nlm.nih.gov/, PRJNA1149528.
