# Supplementary material for: Short-term organic fertilizer substitution increases sorghum yield by improving soil physicochemical characteristics and regulating microbial community structure
Source: Front Plant Sci. 2024 Nov 8;15:1492797. doi: 10.3389/fpls.2024.1492797 (PMC11581943; doi:10.3389/fpls.2024.1492797)
Supplement: Supplementary file 1 [file Table1.docx]

Supplementary Material

# Supplementary Figure
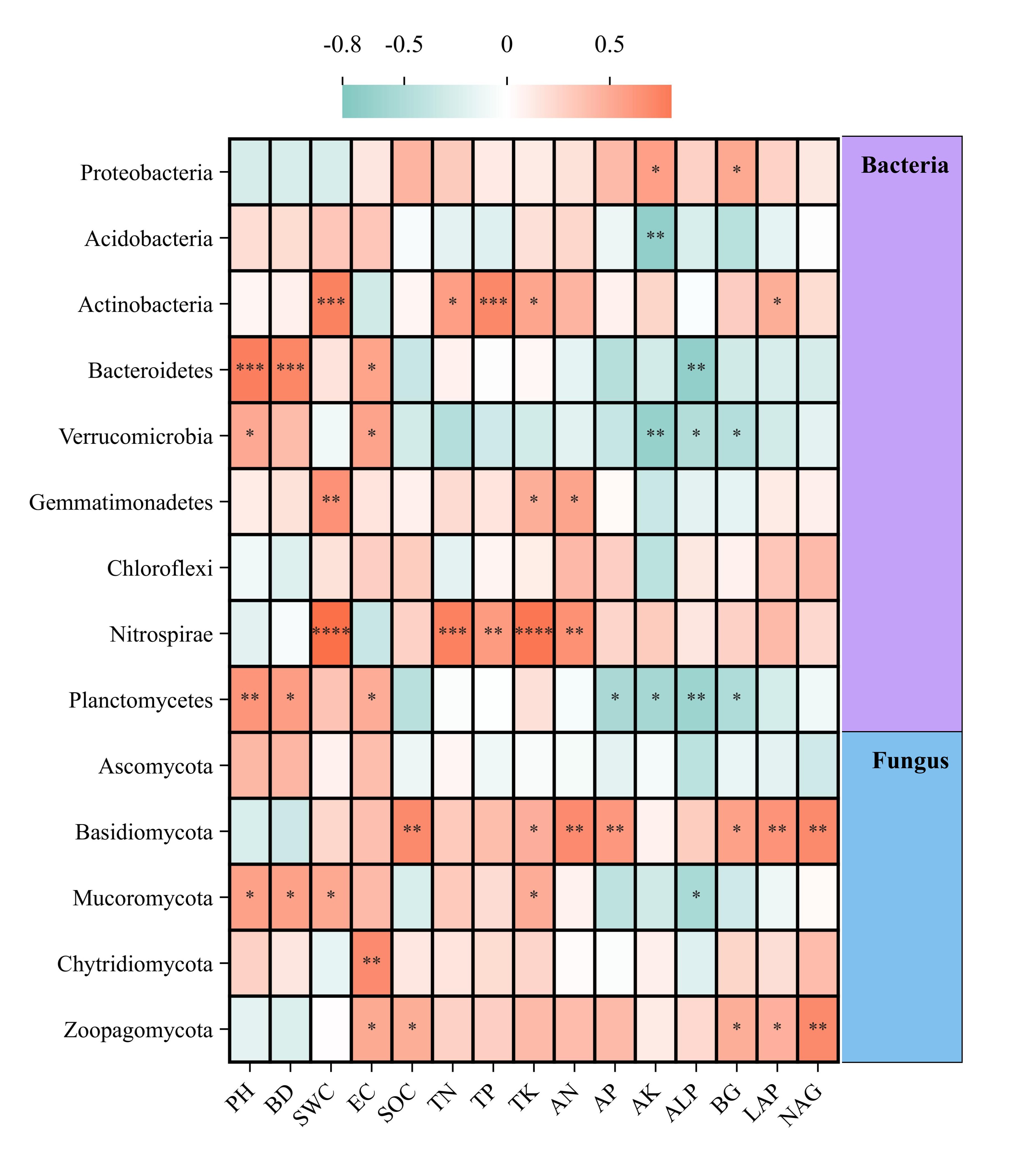


**Supplementary Figure 1.** Spearman correlation analysis heat maps of environmental factors and bacterial and fungal dominant phylum. Red indicates a positive correlation, and blue indicates a negative correlation (* *P* < 0.05; ** *P* < 0.01; *** *P* < 0.001; **** *P* < 0.001).
